# Combined deep-learning MRI-based radiomic models for preoperative risk classification of endometrial endometrioid adenocarcinoma

**DOI:** 10.3389/fonc.2023.1231497

**Published:** 2023-10-16

**Authors:** Jin Yang, Yuying Cao, Fangzhu Zhou, Chengyao Li, Jiabei Lv, Pu Li

**Affiliations:** Clinical School of Obstetrics and Gynecology Center, Tianjin Medical University, Tianjin, China

**Keywords:** endometrial endometrioid adenocarcinoma, MRI, radiomics, preoperative risk classification, deep-learning

## Abstract

**Background:**

Differences exist between high- and low-risk endometrial cancer (EC) in terms of whether lymph node dissection is performed. Factors such as tumor grade, myometrial invasion (MDI), and lymphovascular space invasion (LVSI) in the European Society for Medical Oncology (ESMO), European SocieTy for Radiotherapy & Oncology (ESTRO) and European Society of Gynaecological Oncology (ESGO) guidelines risk classification can often only be accurately assessed postoperatively. The aim of our study was to estimate the risk classification of patients with endometrial endometrioid adenocarcinoma before surgery and offer individualized treatment plans based on their risk classification.

**Methods:**

Clinical information and last preoperative pelvic magnetic resonance imaging (MRI) of patients with postoperative pathologically determined endometrial endometrioid adenocarcinoma were collected retrospectively. The region of interest (ROI) was subsequently plotted in T1-weighted imaging (T1WI), T2-weighted imaging (T2WI), and diffusion-weighted imaging (DWI) MRI scans, and the traditional radiomics features and deep-learning image features were extracted. A final radiomics nomogram model integrating traditional radiomics features, deep learning image features, and clinical information was constructed to distinguish between low- and high-risk patients (based on the 2020 ESMO-ESGO-ESTRO guidelines). The efficacy of the model was evaluated in the training and validation sets of the model.

**Results:**

We finally included 168 patients from January 1, 2020 to July 29, 2021, of which 95 patients in 2021 were classified as the training set and 73 patients in 2020 were classified as the validation set. In the training set, the area under the curve (AUC) of the radiomics nomogram was 0.923 (95%CI: 0.865–0.980) and in the validation set, the AUC of the radiomics nomogram was 0.842 (95%CI: 0.762–0.923). The nomogram had better predictions than both the traditional radiomics model and the deep-learning radiomics model.

**Conclusion:**

MRI-based radiomics models can be useful for preoperative risk classification of patients with endometrial endometrioid adenocarcinoma.

## 1 Introduction

Endometrial cancer (EC) is a malignancy of the inner epithelial lining of the uterus and is the sixth-most commonly occurring cancer among women. EC was diagnosed in 417,367 women in 2020 worldwide, which caused a significant financial burden for patients and carers (1, 2).

It is important to note that Asian women develop endometrial cancer at a younger age than other populations and have more advanced disease, so it is important to classify patients at an early age to manage them suitably (3). Endometrial endometrioid adenocarcinoma is the most common pathological type of EC and can be classified into low-grade (grade I and II) and high-grade (grade III) according to their histological grading (4). It is well known that high-risk factors such as tumor grade, myometrial invasion (MDI), and lymphovascular space invasion (LVSI) play an important role in the choice of procedure and clinical adjuvant therapy as well as prognosis. The ESMO-ESGO-ESTRO guidelines (5) incorporate these factors into the risk classification to guide clinical treatment, which also allows for good identification of the patient’s prognosis (6, 7). Differences in the need for lymph node dissection in high- and low-risk EC patients according to guideline. However, the important factors determining risk classification described above are usually only accurately reported in postoperative pathology, so it is particularly important to identify a non-invasive method that can predict risk classification preoperatively.

Radiomics is a cost-effective and non-invasive approach to characterize tissue intensity, shape, and texture by quantifying the imaging phenotype of the region of interest (ROI) (8–10). Several basic steps are involved, including image acquisition and preprocessing, ROI annotation, feature extraction and selection, and model construction and prediction (11). It aims to link large-scale extracted image information with clinical and biological (12). It can be used not only as a clinical decision tool but also as a research tool for the discovery of new molecular disease pathways (11). These advances in the application of CT, PET, US, and MR imaging can enhance patient stratification and prognostication, thus supporting emerging targeted therapies (12).

Deep learning is a branch of artificial intelligence where networks of simple interconnected units are used to extract patterns from data in order to solve complex problems (13). As described above, radiomics can be a valuable tool for accurate diagnosis and treatment planning. However, ROI segmentation requirements hinder development because the process is too cumbersome and dependent on the experience of the operator. Deep learning algorithms are a good alternative to address this problem because they are capable of automatically learning phenotypic features with powerful characterization capabilities without predefined characteristics and human intervention and are considered advanced radiomics (14–16).

In the field of gynecologic oncology, imaging histology is also widely used for disease diagnosis, prognostic stratification, and treatment strategies (17). Currently published related studies only use traditional radiomic features to construct models. With the development of artificial intelligence and deep learning, we attempted to extract more MRI imaging information for risk prediction by deep-learning methods (18–21). Our study combines traditional radiomics and deep-learning methods to predict the risk classification of endometrial endometrioid adenocarcinoma. To our knowledge, this is the first risk classification model for patients with endometrial endometrioid adenocarcinoma. The aim of our study was to evaluate the patient’s risk classification preoperatively and design individualized treatment plans based on their risk classification.

## 2 Methods

**Figure 1:**
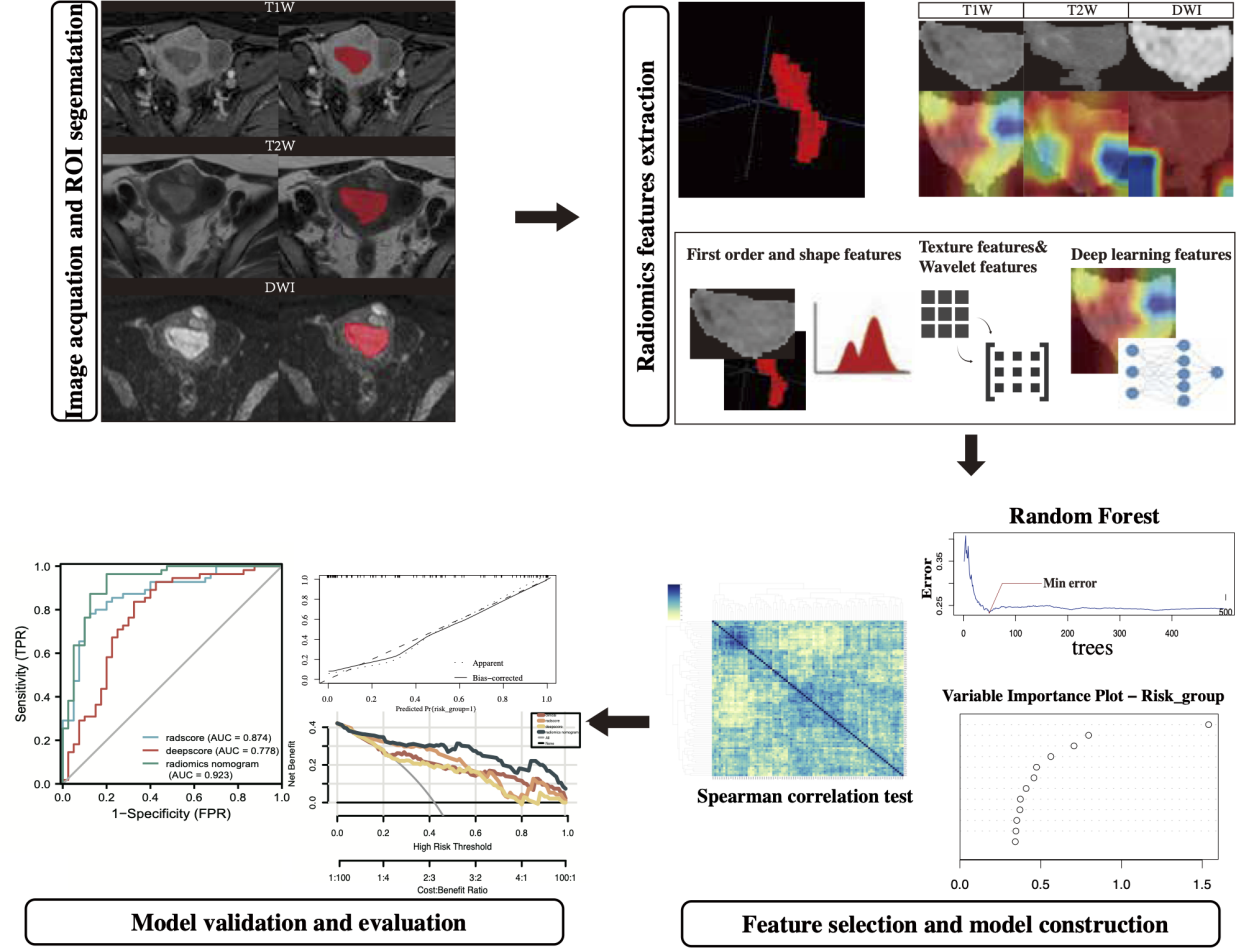
The technology roadmap.

The study was conducted in the following aspects: selection of the population to be included in the study, image acquisition and ROI segmentation, radiomics features extraction, model construction, and validation. (The technology roadmap is shown in **Figure 1**).

### 2.1 Population selection

Patients undergoing endometrial cancer surgery at our hospital from January 1, 2020, to July 29, 2021, were retrospectively selected in the study based on the following criteria. Of these, total hysterectomy + double adnexectomy and lymph node evaluation are the most basic surgical procedures for those with lesions confined to the uterus.

**Figure 2:**
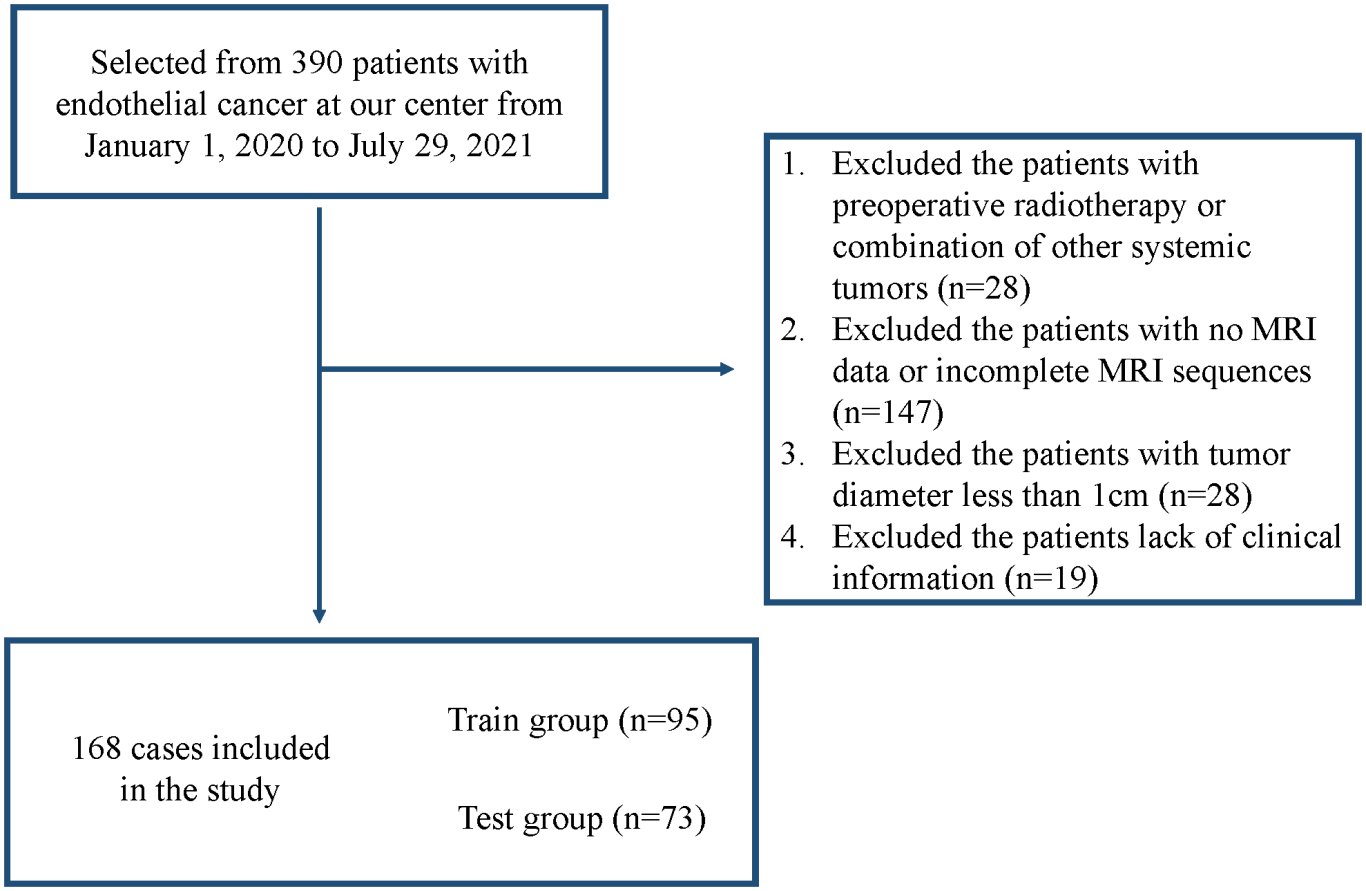
Flowchart of the study population.

Inclusion criteria: (i) all patients were treated surgically without preoperative radiotherapy; (ii) postoperative pathology was reported as endometrial endometrioid adenocarcinoma; (iii) MRI information and clinical information were available at our center; and (iv) T1-weighted imaging (T1WI), T2-weighted imaging (T2WI), and diffusion-weighted imaging (DWI) sequences were complete, and the difference between the time of MRI and the time of surgery was less than 15 days. Exclusion criteria: (i) the patient had other systemic tumors; (ii) the maximum diameter of the tumor presented on MRI was <1 cm; and (iii) the quality of the MRI was poor. **Figure 2** shows the screening flow chart.

In addition, patient’s clinical data including age, CA125 values within 15 days before surgery, tumor size on MRI, tumor grade of the preoperative dilation and curettage (D&C), and family history of first-degree relatives were included. Tumor size was determined by the longitudinal diameter of the largest cross-sectional area of the tumor in the MRI image. Several missing data were filled by the median interpolation method as well as the random interpolation method. Median interpolation was used for CA125 and MRI tumor size, and random interpolation was used for tumor grading of preoperative D&C(fx=RANDBETWEEN).

The final screened patients were divided into a training set and a validation set by year. The risk classification model stratifies patients according to the ESGO/ESMO/ESP 2020 guidelines (molecular typing unknown) into a low-risk (low risk in the risk classification) and a high-risk group (intermediate risk, intermediate-high risk, and high risk are included in the risk classification).

### 2.2 MRI

MRI was performed using a 3.0/1.5 T scanner and an abdominal phased-array coil. Patients were asked to fast for at least 4 h before the examination and to drink water to moderately fill the bladder. The patient was supine and breathing calmly during image acquisition, and the scanning range was from the aortic bifurcation to the lower margin of the symphysis. After gadoteric acid meglumine salt injection was injected intravenously at a rate of 0.2 mmol/kg body weight and 2–3 mL/s, the following sequences were obtained: T1WI, T2WI, and DWI.

### 2.3 Lesion segmentation

**Figure 3:**
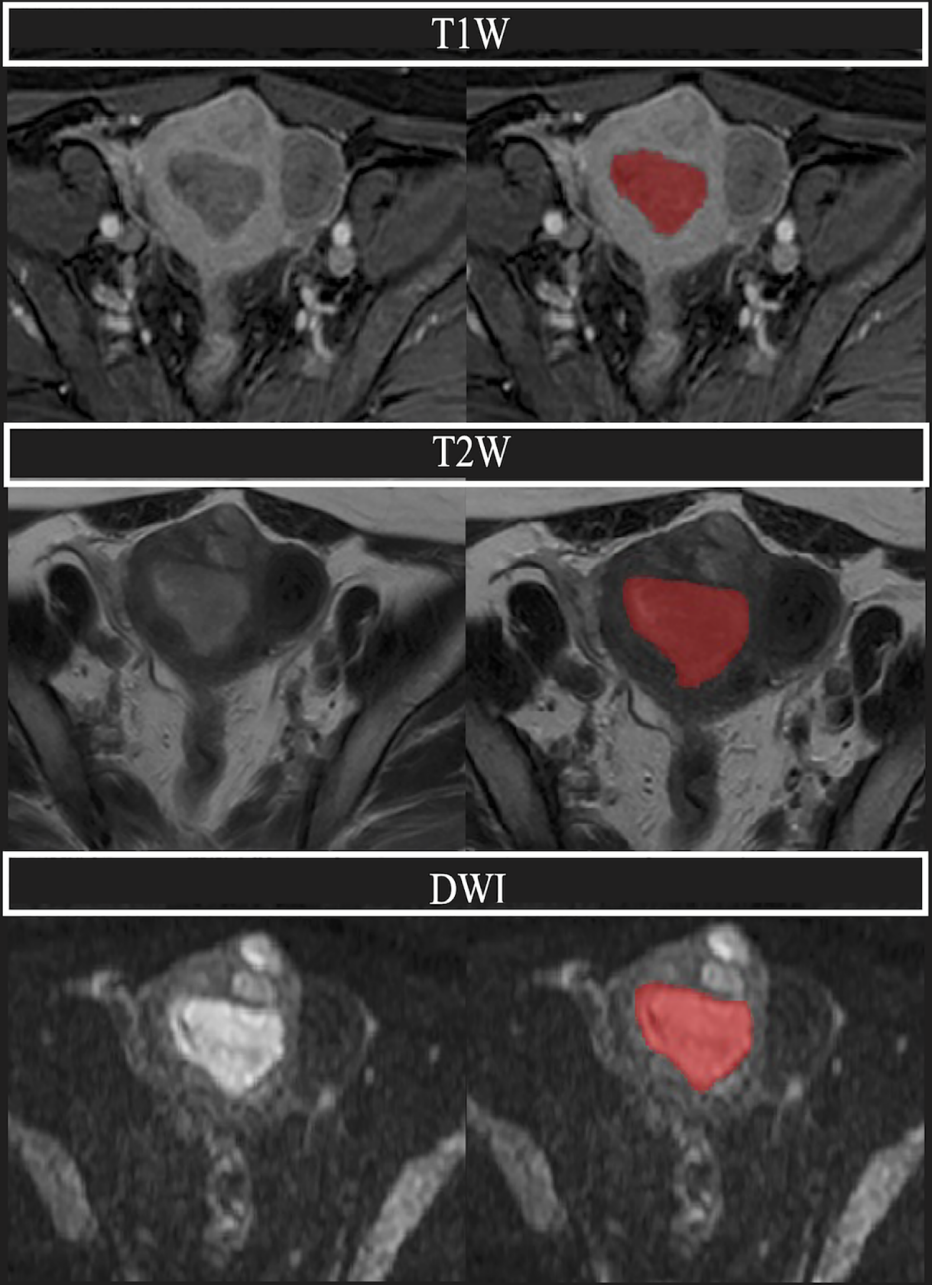
A 68-year-old woman with endometrial adenocarcinoma, classified as intermediate-high risk. Above are the T1W, T2W, and DWI sequences, respectively, and the region covered in red is the plotted ROI (region of interest). We can see that the uterine cavity is replaced by the cancerous foci, and the tumor is lower than the adjacent normal muscle layer in the T1 sequence, while the tumor shows a moderate signal in the T2 sequence, and the tumor shows a significantly high signal in DWI.

All images were exported from our center in Digital Imaging and Communications in Medicine (DICOM) format. The region of interest (ROI) was manually drawn along the edge of the lesion using ITK-SNAP software, layer by layer on T1WI/T2WI/DWI, respectively, and normal tissue was avoided as much as possible to obtain whole-tumor data. The plotting was performed using apparent diffusion coefficient (ADC) sequence contrast to obtain visual support. The tumor showed moderate or low signal intensity on T2WI and high signal intensity on DWI. All ROIs were segmented by three experienced physicians (JY, YC, and FZ), and the final ROIs were determined by mutual agreement among the three physicians. In samples where controversy and disagreement arose during the ROI outlining process, the final opinion was given by another radiologist (HL) with extensive experience. All four physicians were unaware of the clinical and pathologic information of the patients (**Figure 3**).

### 2.4 Radiomics features extraction

Since the MRI scanning machines used in our center are not identical, the images were first preprocessed using N4 bias field correction and then normalized using resampling to unify their voxels to 0.7×0.7×1 mm.

The first-order features; shape features (2D and 3D); grayscale features (Gray Level Concurrence Matrix [GLCM]), gray-level size zone matrix [GLSZM], gray-level run-length matrix [GLRLM], neighborhood gray tone difference matrix [NGTDM] and Gray Level Dependence Matrix [GLDM]); and wavelet features were extracted from the T1, T2, and DWI sequences using the pyradiomics package in Python, respectively, and the number of features extracted from each sequence was 1133. Then, a pre-trained Densenet121 convolutional neural network model was used to identify the maximum cross-section of the ROI for deep-learning image features.

### 2.5 Radiomics feature selection

First, Spearman’s correlation test was performed on the traditional radiomics features extracted from each sequence, and one of the features with correlation coefficients >0.9 was randomly removed for the pairs. The random forest regression model was then used to screen the radiomics features that had a significant impact on risk_group. The random forest regression models were constructed for the radiomics features extracted from DWI sequences, T1 sequences, and T2 sequences, respectively, and the number of trees with the minor mean square error was used as the final number of trees. All features in the models were ranked in terms of importance, and features with importance >0.5 were included in the next step of analysis. For the extracted deep-learning image features, since the features themselves do not have practical meaning, the deep-learning features extracted from each sequence are compressed to 5 in Python (1/20th of the training set sample size).

### 2.6 Model construction and validation

Multinomial logistic regression was used to construct a multi-sequence traditional radiomics signature on the screened traditional radiomics features in the training set. The traditional radiomics risk score (radscore) was calculated for each sample based on the predicted function of the ROCR package. Meanwhile, the deep-learning radiomics risk score (deepscore) was calculated for each sample by using the same method. Radscore and deepscore were then used as variables along with the patient’s clinical information (age, CA125 values within 15 days before surgery, tumor size on MRI, tumor grade of D&C, and family history of first-degree relatives) to construct a nomogram predicting risk_group (called radiomics nomogram). Internal validation of the model was carried out using bootstrap method. Validation of the nomogram used data from the validation set.

### 2.7 Evaluation of radiomics nomogram

The ROC curve, calibration curve, decision curve analysis (DCA) curve, sensitivity, specificity, positive predictive value, negative predictive value, Youden index, and prediction accuracy of the training set were plotted. The cut-off value of the model according to the ROC curve and Youden index was calculated, and the risk of the samples in the validation set according to this cut-off value was graded, followed by further plotting the ROC curve and DCA curve of the model in the validation set. Based on the prediction results, various evaluation indices of the model were further calculated.

### 2.8 Statistical analysis

The ITK-SNAP used in this study was version 3.8.0 and the Python used was version 3.7.0. Continuous variables in the baseline profile were expressed as median values (IQR) because they did not satisfy normal distribution. For model validation using data from the validation set, each data in the validation set was assigned a value of 0 or 1 based on the cut-off value obtained from the ROC of the training set, and then the risk score was calculated. The variability between the different models was compared using the Delong test. For all statistical analyses, a p-value less than 0.05 was considered statistically significant. All statistical analyses were done using R software (Version 4.2.1).

## 3 Results

### 3.1 Patients

From January 1, 2020, to July 29, 2021, a total of 390 patients underwent surgical treatment for EC at our institution, and 222 patients were excluded according to the inclusion and exclusion criteria, resulting in 168 patients being finally included in the study (95 in the training set [2021] and 73 in the validation set [2020]).

All clinical and pathological information is presented in **Table 1**. The number of missing CA125 was 13, the number of missing MRI tumor size was 10, and the number of missing D&C grade was 15. The value of CA125 was 17.74 according to the median, the value of MRI tumor size was 3.3 cm according to the median, and the D&C grade was randomly interpolated from 0 and 1.

**Table 1:** Clinical and pathological information of patients with endometrial endometrioid adenocarcinoma included in the study, where age, CA125, MRI tumor size, D&Cgrade, and first-degree family tumor history were finally included in the radiomics nomogram.

Variable	Overall N = 168	Train N = 95	Validation N = 73	p-value
**Age (IQR)**	56 (51, 62)	56 (50, 62)	56 (51, 62)	0.6
**CA125 (IQR)**	17 (12, 26)	16 (12, 25)	18 (12, 28)	0.4
**Tumor size (IQR)**	3.20 (2.48, 4.30)	3.20 (2.50, 4.25)	3.30 (2.40, 4.30)	0.6
**D&C grade (%)**				0.035
**G1+G2** **G3**	146(87%) 22 (13%)	78(82%) 17 (18%)	68(93%) 5 (7%)	
**Family history(%)**				>0.9
**(+)** **(–)**	23(14%) 145(86%)	13 (14%) 82(86%)	10 (14%) 63(86%)	
**FIGO** **IA** **IB** **II** **III-IV** **LVSI** **(+)** **(–)** **MDI** **> 50%** **< 50%** **Risk group(%)** **Low** High* Intermediate intermediate-high High	125(74%) 17(10%) 7(4%) 19(11%) 54(32%) 114(68%) 33(20%) 135(80%) 94(56%) 74(44%) 13(8%) 42(25%) 19(11%)	74 (78%) 7 (7%) 4 (4%) 10 (11%) 25 (26%) 70 (74%) 12(13%) 83(87%) 55(58%) 40 (42%) 9(10%) 20(21%) 11(12%)	51(70%) 10(14%) 3(4%) 9(12%) 29(40%) 44(60%) 21(29%) 52(71%) 39(53%) 34 (47%) 4(5%) 22(30%) 8(11%)	0.6

Among the 95 patients in the training set, tumor grade G1+G2: 78 cases (82%) and grade G3: 17 cases (18%); FIGO stage IA: 74 cases (78%), stage IB: 7 cases (7%), stage II: 4 cases (4%), stage III–IV: 10 cases (11%); LVSI positive: 25 cases (26%) and LVSI negative: 70 cases (74%); myeloid infiltration depth >50%: 12 cases (13%), myeloid infiltration depth <50%: 83 (87%); risk stratification was low risk in 55 cases (58%), intermediate risk in 9 cases (10%), intermediate-high risk in 20 cases (21%), and high risk in 11 cases (12%).

Of the 73 patients in the validation set, tumor grade G1+G2: 68 cases (93%) and grade G3: 5 cases (7%); FIGO stage IA: 51 cases (70%), stage IB: 10 cases (14%), stage II: 3 cases (4%), stage III–IV:9 cases (12%); LVSI positive: 29 cases (40%), LVSI negative: 44 cases (60%); myeloid infiltration depth >50%: 21 cases (29%), myeloid infiltration depth <50%: 52 cases (71%); risk stratification was low risk in 39 cases (53%), intermediate risk in four cases (5%), intermediate-high risk in 22 cases (30%), and high risk in eight cases (11%) (**Table 1**).

### 3.2 The extracted radiomics features and the established model

Each series extracted 1133 traditional radiomics features, and after Spearman’s correlation test and random forest regression, the number of trees with the smallest mean square error was selected, and then the features were ranked in terms of variable importance. The number of features with variable importance >0.5 were: two for the T1(log.sigma.5.0.mm.3D_firstorder_Maximum%wavelet.LHH_firstorder_RootMeanSquared), six for T2(wavelet.HLL_gldm_DependenceVariance&log.sigma.3.0.mm.3D_glcm_InverseVariance&log.sigma.4.0.mm.3D_glszm_SmallAreaEmphasis&wavelet.LLL_glszm_ZoneVariance&wavelet.HHL_glcm_Imc2&wavelet.LHH_gldm_SmallDependenceLowGrayLevelEmphasis), and four for DWI(wavelet.LLL_glcm_Correlation&wavelet.LLL_firstorder_Minimum&original_shape_Flatness%wavelet.HHH_firstorder_Kurtosis).

**Figure 4:**
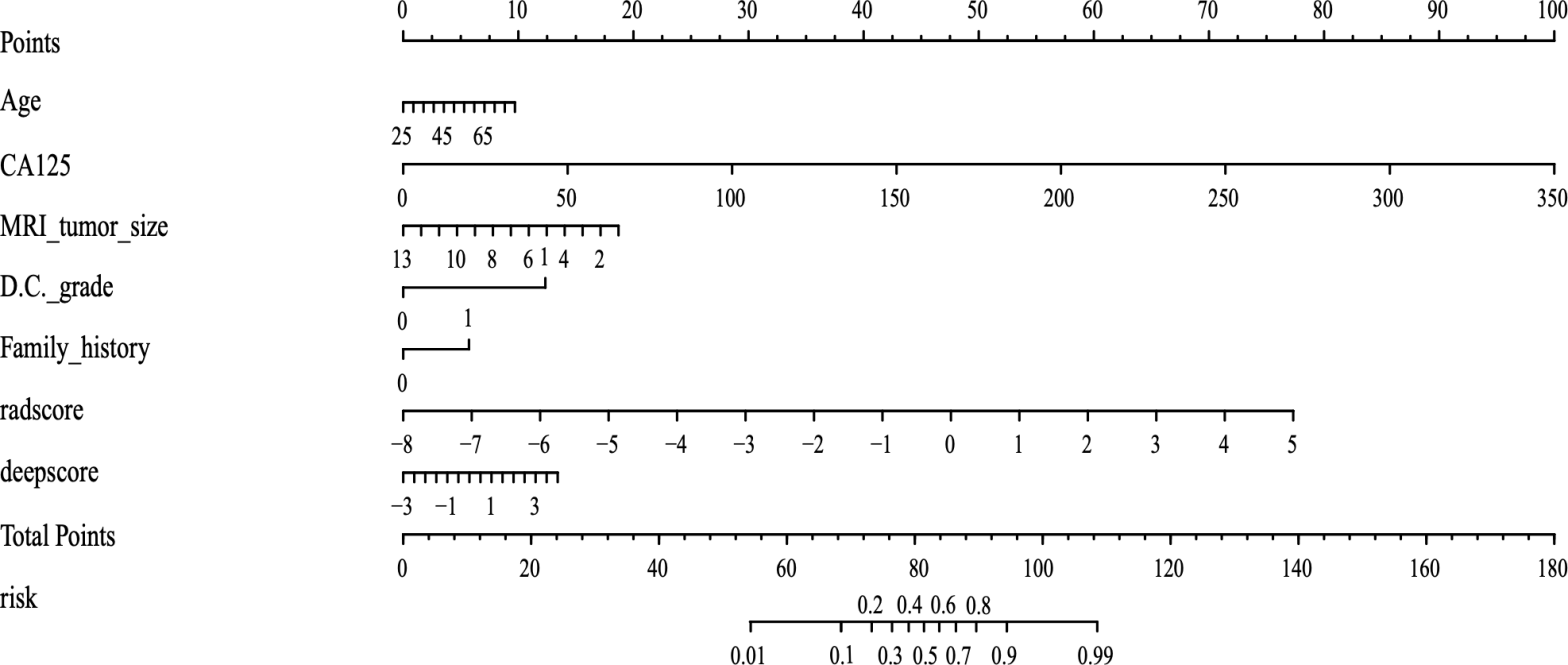
The final radiomics nomogram, which includes three parts: radscore, deepscore, and clinical information. The different values correspond to different scores on the straight line, and the total score finally corresponds to the predicted value.

The deep learning features extracted from each sequence were compressed to 5 in Python. The final radiomics nomogram model combines the radscore with the deepscore and clinical information (including age, CA125 values within 15 days before surgery, tumor size on MRI, tumor grade of D&C, and family history of first-degree relatives) (**Figure 4**).

### 3.3 Diagnostic performance of the radiomics nomogram

**Figure 5:**
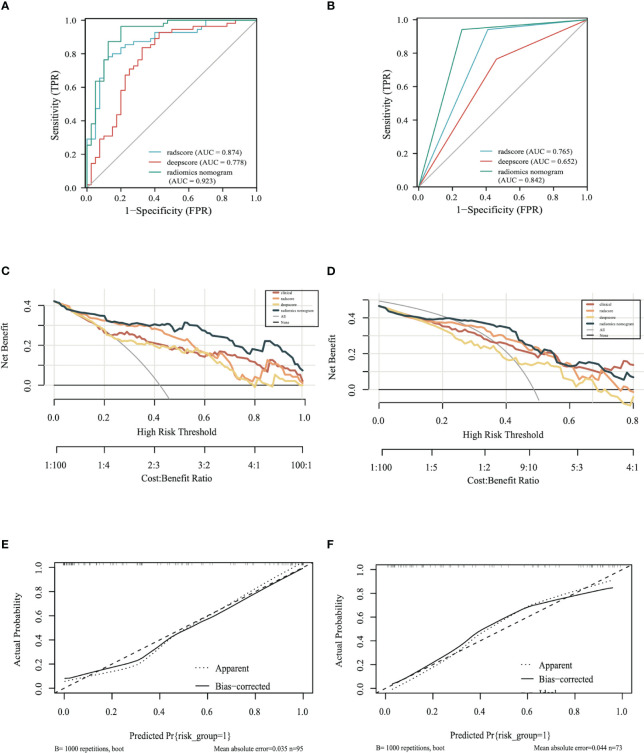
Model evaluation: **(A)** ROC of the models in training set **(B)** ROC of the models in the validation set. **(C)** The decision curve of the training set. **(D)** The decision curve of the validation set. **(E)** Calibration curve of the radiomics nomogram of the training set. **(F)** Calibration curve of the radiomics nomogram of the validation set.

In the training set, the area under the curve (AUC) of the radscore was 0.874, the AUC of the deepscore was 0.778, and the AUC of the radiomics nomogram was 0.923 (**Figure 5A**). The mean value of Dxy’s high valuation of nomogram obtained after 1000 resamples of internal validation was 0.058, and the corrected mean value of Dxy was 0.787. The C-index of internal validation was calculated to be 0.894. After risk grading the validation set according to the cut-off values, the calculated AUCs were 0.765 for radscore, 0.652 for deepscore, and 0.842 for the radiomics nomogram (**Figure 5B**). The decision curves showed that radiomics nomogram had the best clinical net benefit in both the training and validation sets (**Figures 5C, D**). The calibration curve for the training set showed good calibration performance (mean square error, mse=0.00184) (**Figure 5E**). The calibration curve for the validation set also showed good calibration performance(mean square error, mse=0.00302) (**Figure 5F**).

The AUC/sensitivity/specificity/PPV/NPV and Youden Index are shown in **Table 2**.

**Table 2:** Prediction performance of radscore, deepscore, and radiomics nomogram models in the training and validation sets.

Model	AUC(CI)	Sensitivity	Specificity	PPV	NPV	Youden Index
Train set						
**Tra_rad**	0.874(0.802-0.946)	0.764	0.900	0.913	0.735	0.664
**Deep_rad**	0.778(0.677-0.879)	0.836	0.675	0.780	0.750	0.511
**Final_rad**	0.923(0.865-0.980)	0.964	0.800	0.869	0.941	0.764
Test set						
**Tra_rad**	0.765( 0.678-0.853)	0.941	0.590	0.667	0.920	0.531
**Deep_rad**	0.652( 0.544-0.759)	0.765	0.538	0.591	0.724	0.303
**Final_rad**	0.842(0.762-0.923)	0.941	0.744	0.762	0.935	0.685

The results of the Delong test in the training set showed that the final radiomics nomogram outperformed the radscore (P=0.037) and deepscore (P=0.001) in predicting 0 and 1 outcomes, with statistically significant results; the results in the validation set also showed that the radiomics nomogram outperformed the radscore (P=0.037) and deepscore (P=0.001) in predicting 0 and 1 outcomes. The results in the validation set also showed that the diagnostic efficacy of the radiomics nomogram was better than radscore (P=0.037) and deepscore (P=0.001), and the results were statistically significant.

## 4 Discussion

This study combines the features of traditional radiomics with those of deep learning. In terms of preoperative prediction of risk classification for endometrial endometrioid adenocarcinoma, our results show that MRI-based radiomics nomogram has good diagnostic efficacy. Our study also included clinical information such as the patient’s age, CA125, MRI tumor size, D&C grade, and first-degree family tumor history, which are often considered independent influencing factors for high-risk EC (22–24). With the exception of the D&C grade (P=0.035), the rest of the indicators were not statistically significant in either the training or the validation sets. The percentage of D&C grade in G3 in the training set was 18%, while that in the validation set was 6.8%. Our analysis suggests that the preoperative D&C of the population included in the study was not exactly performed in the same center and that individual differences in the reading of the films by different pathologists in different centers may occur. Moreover, the base of the population we included in the study was not large enough to offset this variability.

Endometrial endometrioid adenocarcinoma is the most common type of pathology; however, to date, no model has been developed that can predict the risk classification of endometrial endometrioid adenocarcinoma. Previously, some scholars developed models for risk classification of EC, for example, the study by Moro et al. was based on a model constructed by MRI as well as ultrasound, and they ended up with an AUC value of 0.85 in the validation set, which is similar to the results of our model (25). Bi Cong et al. also developed a radiomics-based nomogram to predict risk classification and found that this model was approximately 11–15% more beneficial than actual surgery in the surgical management of patients (26). Celli et al. also developed a model to preoperatively predict the presence of LVSI and risk classification of patients with EC based on radiomics, differing from the previous two studies, in that he used a risk classification based on molecular typing (27). Kaiyue et al. developed a nomogram combining ADC values as well as radiomics for preoperative prediction of high-risk factors such as EC-grade, DMI, and LVSI (28). In addition, other similar studies have been conducted (29, 30).

Wang Y et al. also established a preoperative model for predicting whether patients with endometrial endometrioid adenocarcinoma have deep myometrial infiltration based on imaging histology (31). Yan B et al. established an imaging histology model for preoperative prediction of LVSI for patients with endometrial endometrioid adenocarcinoma by combining the images of the tumor and peritumoral region (32). The above two studies mainly decided the surgical program (i.e., whether lymph node dissection was needed) by predicting whether there was deep myometrial infiltration and whether there was LVSI positivity, whereas the risk stratification in the ESGO/ESMO/ESP 2020 guidelines utilized in our article incorporates comprehensive pathological information that includes deep myometrial infiltration, LVSI, and so on, and this risk stratification determines the patient’s surgical approach. Therefore, our predicted risk stratification is more comprehensive and convincing than the direct prediction of a single piece of information.

The risk stratification of the ESMO guidelines contains three main aspects: LVSI, FIGO staging, and histologic type. Many studies have confirmed that LVSI plays an extremely important role in the prognosis of patients with endometrial cancer. For example, Restaino S et al. (33) performed a semi-quantitative analysis of LVSI in EC patients and found that diffuse LVSI was the strongest independent prognostic factor for lymph node metastasis and distant metastasis. In addition, they found diffuse LVSI to be associated with a higher risk of recurrence and decreased OS. Tortorella L et al. (34) focused on patients with early-stage, low-risk endometrial carcinoma and assessed, in a semi-quantitative manner, the prognostic impact of LVSI and showed that Substantial LVSI represents the strongest independent risk factor for decreased survival and distant relapse. According to the risk classification of the ESMO guidelines (6), the lowest possible risk of non-endometrial endometrioid adenocarcinoma is “intermediate”, and the guidelines also state that surgical staging or lymph node biopsy or resection should be considered for intermediate risk or higher, but not for low-risk patients. The type of pathology is confirmed by preoperative D&C, and it is highly consistent with postoperative pathology (35). Therefore, we believe it is necessary to create a separate risk classification model for patients with endometrial endometrioid adenocarcinoma.

In conclusion, the radiomics nomogram can predict risk classification of endometrial endometrioid adenocarcinoma preoperatively with high efficacy, which can help clinicians to develop individualized treatment plans. The strengths of our study are as follows: first, we used methods that combine traditional radiomics with deep-learning to extract features. Second, to our knowledge, this is the first study to address risk classification of endometrial endometrioid adenocarcinoma. Third, we included external validation and achieved good diagnostic efficacy in the external validation set.

This study also has some limitations. First, this was a single-center retrospective study that included only patients who underwent surgery in our center, which may have caused selection bias and lack of power than other multicenter studies. Second, there were some missing clinical data in this study, and although interpolation was performed, the diagnostic efficacy of the final nomogram might have been better if the clinical information had been complete.

## 5 Conclusion

This study combines traditional radiomics and deep learning of image features to develop a model that predicts risk classification of endometrial endometrioid adenocarcinoma preoperatively to help clinicians develop individualized treatment plans. To our knowledge, this is the first predictive model for patients with endometrial endometrioid adenocarcinoma.

## Data availability statement

The raw data supporting the conclusions of this article will be made available by the authors, without undue reservation.

## Ethics statement

The studies involving humans were approved by Ethics Committee of Tianjin Central Hospital of Obstetrics and Gynecology. The studies were conducted in accordance with the local legislation and institutional requirements. Written informed consent for participation was not required from the participants or the participants’ legal guardians/next of kin in accordance with the national legislation and institutional requirements.

## Author contributions

JY and PL designed the study. YC and FZ performed the research. CL provided help and advice on the acquisition of data. JL analyzed the data. JY wrote the manuscript. All authors contributed to the article and approved the submitted version.
